# Stand-Alone CuFeSe_2_ (Eskebornite) Nanosheets for Photothermal Cancer Therapy

**DOI:** 10.3390/nano11082008

**Published:** 2021-08-05

**Authors:** Mimi Liu, Daniela R. Radu, Gurpreet Singh Selopal, Saiphaneendra Bachu, Cheng-Yu Lai

**Affiliations:** 1 Department of Mechanical and Materials Engineering, Florida International University, Miami, FL 33199, USA; mliu@fiu.edu (M.L.); dradu@fiu.edu (D.R.R.); 2 Centre Énergie Materiaux Télecommunications (INRS-EMT), Institut National de la Recherche Scientifique, Varennes, QC J3X 1S2, Canada; gurpreet.selopal@inrs.ca; 3 Department of Materials Science and Engineering, The Pennsylvania State University, University Park, PA 16802, USA; sbachu@psu.edu

**Keywords:** CuFeSe_2_ nanosheets, doxorubicin, cytotoxicity, photothermal therapeutic efficacy

## Abstract

Two-dimensional CuFeSe_2_ nanosheets have been successfully obtained via solution-phase synthesis using a sacrificial template method. The high purity was confirmed by X-ray diffraction and the two-dimensional morphology was validated by transmission electron microscopy. The intense absorption in the 400–1400 nm region has been the basis for the CuFeSe_2_ nanosheets’ photothermal capabilities testing. The colloidal CuFeSe_2_ (CFS) nanosheets capped with S^2−^ short ligands (CFS-S) exhibit excellent biocompatibility in cell culture studies and strong photothermal effects upon 808 nm laser irradiation. The nanosheets were further loaded with the cancer drug doxorubicin and exposed to laser irradiation, which accelerated the release of doxorubicin, achieving synergy in the therapeutic effect.

## 1. Introduction

CuFeSe_2_, a ternary chalcogenide in the I–III–VI_2_ group, features a narrow bandgap of 0.16 eV in bulk [1]. The material has a metallic character at room temperature; however, in nanoparticle form, it shows a direct optical band gap energy of 0.95 eV, [1] much wider than the bulk CuFeSe_2_, and this enables applications in cancer phototherapy, which requires a near-infrared (NIR) absorber.

Several reports relay that CuFeSe_2_ nanoparticles are biocompatible and effective as photothermal agents. CuFeSe_2_ nanoparticles with a 5 nm diameter reported recently exhibit good biocompatibility, high photothermal conversion efficiency, and colloidal stability [2]. The deposition of CuFeSe_2_ nanocrystals in bioactive glass scaffolds (BG-CFS) led to composites with excellent photothermal performance, which ablate bone tumor cells in vitro and significantly inhibit bone tumor tissue growth in vivo [3]. Lai et al. demonstrated that the CuFeSe_2_@diethylenetriaminepenta acetic acid (DTPA)-Gd nanomaterial is adequate for CT and MRI T1WI/T2WI three-mode imaging, and could be employed as a multimodal contrast agent [4]. Recently, the unique optoelectronic properties and chemical resilience of two-dimensional (2D) materials opened new horizons regarding their use in optoelectronics, energy generation and storage, sensing, and biomedical applications, including cancer phototherapy [5,6,7,8,9,10,11,12,13]. A 2D morphology is highly desired in phototherapy, since 2D materials’ increased light absorption enhances their ability to generate heat, while their large surface area and atomic thickness increase their loading capacity for drugs or biomacromolecules [7,14,15]. A high photothermal conversion efficiency and anticancer efficacy were reported for 70 nm CuFeSe_2_ nanosheets [16]. Hierarchical assemblies of CuFeSe_2_ nanosheets, prepared through a cation exchange method, were recently reported [17].

In this study, 2D CuFeSe_2_ nanosheets have been successfully obtained via solution-phase synthesis using a sacrificial template method. Synchrotron X-ray characterization and Raman spectroscopy show high material purity. The as-synthesized CuFeSe_2_ nanosheets were subjected to a ligand exchange process, leading to CuFeSe_2_ nanosheets capped with S^2−^ (CFS-S). In vitro toxicity tests on two different cell lines demonstrated the excellent biocompatibility of the capped-nanosheets. Despite the good photothermal effect, previous reports utilized polymer-coated CFS nanosheets, which are further functionalized to facilitate drug loading [16]. Herein, the CFS-S nanosheets are stand-alone and readily dispersible, and are thus suitable to be administered directly into tumors. A remarkable decrease in survival rate for cancer cells was observed when the cells treated with CFS-S nanosheets were exposed to 808 nm NIR laser irradiation, demonstrating CFS-S’s photothermal effect. Upon treatment with doxorubicin-loaded CFS-S (CFS-S-DOX), a synergistic therapeutic effect was measured. Each photothermal therapy study entailed corresponding control experiments, including cells treated with CFS-S without irradiation, free DOX treatment control, and CFS-S-DOX cells treated without laser irradiation. 

## 2. Materials and Methods

### 2.1. Materials

All chemicals used in this work were used as received, without further purification. Copper(II) chloride dihydrate (CuCl_2_·2H_2_O, 99.999, 1-dodecanethiol (1-DDT, ≥98%), Fe(III) 2,4-pentanedionate (Fe(acac)_3_, 97%), oleylamine (OLA, 70%), and selenium (99%) were purchased from Sigma-Aldrich (Saint Louis, MI, USA); ACS grade chloroform (99.8%) and ethanol (99.5%) from VWR International (Radnor, PA, USA); sodium sulfide (Na_2_S, anhydrous) and formamide (FA, 99%) from Alfa Aesar (Tewksbury, MA, USA), ACS-reagent-grade nanopure water from Ricca chemical company (Arlington, TX, USA), and doxorubicin hydrochloride salt (DOX, >99%) from LC Laboratories (Woburn, MA, USA). Cells Counting kit-8 (CCK-8) was purchased from GLPBIO Technology Inc (Montclair, CA, USA). The Gibco Cell Culture Media and F-12K nutrient mixture were bought from Thermo Fisher (Waltham, MA, USA), and Dulbecco’s phosphate-buffered saline (DPBS) from Lonza Bioscience (Walkersville, MD, USA). The HeLa cervical cancer cells were purchased from the Antibody Research Corporation (Saint Charles, MO, USA), and the Green Fluorescence Protein (GFP)-modified lung cancer cells (A549GFP) from MyBioSource, Inc (San Diego, CA, USA). 

### 2.2. Preparation of CuFeSe_2_ Nanosheets

The synthetic pathway involves the formation of templating FeSe_2_ nanosheets through a reported procedure [8], followed by the addition in situ of a copper precursor. In a typical synthesis, copper(II) chloride dihydrate (0.67 mmol, 114 mg) and 5 mL OLA were added to a 25 mL glass flask to prepare the Cu precursor. Separately, Fe(III) 2,4-pentanedionate (0.5 mmol, 177 mg) and 10 mL of OLA were loaded into a 100 mL 2-neck round-bottom quartz flask to obtain the Fe precursor. Se precursor was prepared by combining 1 mmol selenium (78.96 mg) with 2.5 mL of 1-DDT in 3 mL of OLA. Both Fe and Se precursors were stirred at room temperature for 30 min under vacuum, while the Cu precursor was degassed at 100 °C for 30 min. The dark-brown Fe precursor was heated to 140 °C under argon atmosphere, followed by the immediate injection of the Se precursor. The reaction mixture was heated to 220 °C and kept at the same temperature for 30 min to form FeSe_2_ nanosheets. Afterward, the Cu precursor was rapidly injected into the formed FeSe_2_ solution and then held at 220 °C for 1 h. The reaction mixture was cooled to room temperature by removing the heating source. To purify the product, first, OLA (8 mL) and 1-DDT (1 mL) were added to remove residual Se [8]. Second, a mixture of chloroform and ethanol (V:V, 1:3) was used to wash the CuFeSe_2_ nanosheets (NSs) product; this step was repeated three times. The precipitate was dried overnight under vacuum. 

### 2.3. Functionalization of CuFeSe_2_ NSs with S^2−^ (Ligand Exchange)

In total, 20 mL of CuFeSe_2_ NS as a chloroform suspension (5 mg/mL) was mixed with a 20 mL Na_2_S–formamide solution (10 mg/mL) and stirred until the dark CuFeSe_2_ NSs transferred to the formamide phase. The resulting transparent chloroform layer was removed and the dark CuFeSe_2_ capped with S^2−^ (CFS-S) was precipitated and washed three times with 40 mL of ethanol and nanopure water (V:V, 1:1). The final product was dried under vacuum. 

### 2.4. Characterization

X-ray diffraction (XRD) measurement of the product were performed on a Rigaku MiniFlex600 (Rigaku, Tokyo, Japan) equipped with Cu Kα radiation (λ = 1.5405 Å). A WITec alpha 300 Raman spectroscope (WITec, Ulm, Germany) equipped with an Ar laser source (λ = 532 nm) was used to confirm the structure of the prepared FeSe_2_ and CuFeSe_2_ powders. Transmission electron microscopy (TEM) images were obtained on a Thermo Fisher Talos F200x scanning/transmission electron microscope (S/TEM) (Thermo Fisher Scientific, Waltham, MA, USA) to investigate the morphology of the product. A field emission scanning electron microscope assembled with energy dispersive X-ray spectroscopy (SEM-EDS) (JEOL 6330F, Peabody, MA, USA) was used to determine the morphology and elemental distribution of the prepared CuFeSe_2_ nanosheets and CFS-S. The redox states of elements for the prepared CuFeSe_2_ nanosheets and CFS-S were determined by X-ray photoelectron spectroscopy (XPS) in a VG Escalab 220i-XL (VG Scientific, Waltham, MA, USA) equipped with an Al Kα source. The UV–Vis–NIR spectra of CuFeSe_2_ nanosheets were collected using a UV-3600 plus spectrophotometer (Shimadzu, Kyoto, Japan). The thermal stability of the CuFeSe_2_ nanosheets was determined using a TA Instrument SDT-Q600 (TA Instruments, New Castle, DE, USA). Dynamic light scattering (DLS, Malvern Panalytical, Malvern, UK) was used to determine the hydrodynamic diameter, size distribution and zeta potential of CFS-S and CFS-S-DOX. An 808 nm NIR laser (RLDH808–1200-5, Roithner Laserthchnik Gmbh, Vienna, Austria) was used for the photothermal therapy study. The temperature of the solution was recorded with a TH-5 Thermalert Clinical Monitoring Thermometer (Physitemp Instruments, Clifton, NJ, USA). A heated stage insert (World Precision Instruments Inc.) was used to hold the cell culture plates at 37 °C. 

### 2.5. Photothermal Effect

To explore the photothermal effect of CFS-S, a series of CFS-S aqueous solutions (250 µL) with concentrations from 2.5 µg/mL to 160 µg/mL were irradiated with an 808 nm NIR laser with an output power density of 1 W/cm^2^ for 10 min. The temperature of each solution was measured by a thermocouple linked to a digital thermometer and recorded every 10 s. Additionally, a CFS-S aqueous solution with a fixed concentration of 20 µg/mL was irradiated by an 808 nm NIR laser for 10 min over five on–off cycles to investigate the photothermal stability of the materials. The photothermal conversion efficiency of the CFS-S was evaluated using a reported equation (Supplementary Materials) [18,19].

### 2.6. Drug Loading

The 4 mg CFS-S nanosheets were dispersed into 4 mL nanopure water using probe sonication, then mixed with the chemotherapy drug doxorubicin (DOX) in different ratios. The mixture was stirred at room temperature for 24 h and then centrifuged to collect the precipitates and supernatant. After centrifugation and washing with nanopure water, the precipitates were dispersed into 4 mL of nanopure water to obtain the CFS-S-DOX (1 mg/mL) stock solution. Meanwhile, the DOX in the supernatant was investigated by UV–Vis–NIR spectroscopy to evaluate the amount of unloaded DOX, so as to determine the optimal ratio of CFS-S to DOX, and the loading efficiency was calculated according to the literature (SI) [20].

### 2.7. In Vitro Drug Release

In a typical measurement, 4 mL of CFS-S-DOX aqueous solution (400 μg/mL) was irradiated with an 808 nm NIR laser (1 W/cm^2^) for 10 min. Meanwhile, an equal volume of the 400 μg/mL CFS-S-DOX aqueous solution was held at room temperature without laser irradiation. The amount of free DOX in these two solutions was determined by UV-Vis spectroscopy at intervals of 1 h, 2 h, 4 h, 24 h, and 48 h after irradiating with an NIR laser.

### 2.8. Cell Culture and Cytotoxicity of CFS-S In Vitro

The in vitro cytotoxicity of CFS-S was assessed using the Cell Counting Kit-8 (CCK-8), GLPBIO Technology Inc. (Montclair, CA, USA). in HeLa and A549GFP cells. Cells were seeded into 96 well plates (8000 cells/well) in Ham’s F-12K medium at 37 °C and in a 5% CO_2_ atmosphere for 24 h. Then, 25 μL of CFS-S aqueous dispersion at different concentrations (0, 2.5, 5, 10, 20, 40, 80, 160, 200 μg/mL) was added into each well, which contained 225 μL fresh medium. After an additional 24 h of incubation at 37 °C, the culture CFS-S-containing medium was replaced with 10 μL CCK-8 in 100 μL fresh medium, and each well absorbance was measured (450 nm) in five replicates for each concentration.

### 2.9. In Vitro Photothermal Ablation of Cancer Cells

HeLa and A549GFP cells were seeded into 96-well plates (8000 cells/well), followed by incubation at 37 °C for 24 h, and then we replaced the culture medium with a mixture of 225 μL fresh medium and 25 μL CFS-S or CFS-S-DOX solution at 0, 2.5, 5, 10, 20 and 40 μg/mL. The plate was incubated at 37 °C for 2 h to allow the drug solution to mix, and then subjected to laser treatment for 10 min, followed by an additional 24 h incubation (37 °C). The cell viability was evaluated by the CCK-8 assay. Each treatment was done in 5 replicates.

## 3. Results and Discussion

FeSe_2_ nanosheets, prepared through a reported procedure [8], show a diffraction pattern corresponding to an orthorhombic FeSe_2_ structure (PDF#: 65-2570, Space group: Pnnm) (SI, Figure S1a). Raman spectroscopy (Figure S1b) validated the compound’s identity. The FeSe_2_ (TEM image, Figure S1c) is ultrathin and exhibits a large lateral size of 700–800 nm.

**Figure 1:**
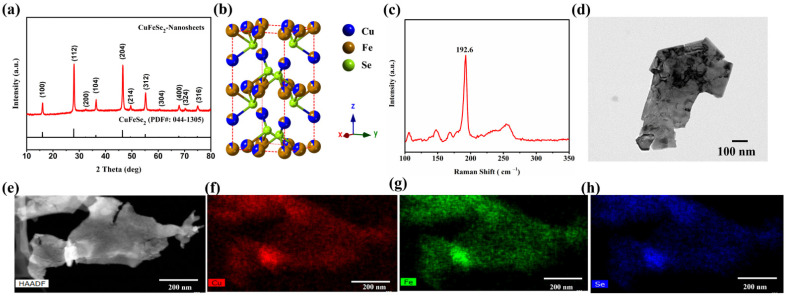
(**a**) XRD pattern. (**b**) Crystal structure. (**c**) Raman spectrum. (**d**) TEM images of the synthesized CuFeSe_2_ nanosheets. (**e**–**h**) STEM-EDS of the synthesized CuFeSe_2_ nanosheets.

The CuFeSe_2_ nanosheets derived from the formed FeSe_2_ nanosheets have a tetragonal structure (space group $P\bar{4}2c$, a = 5.521 Å, c = 11.026 Å) confirmed by XRD (Figure 1a), and maintain the 2D morphology of the FeSe_2_ template (Figure 1d). The full XRD pattern of as-synthesized CuFeSe_2_ nanosheets in Figure S2 measured by synchrotron X-ray also corresponds to the tetragonal structure of the space group $P\bar{4}2c$, showing the excellent purity of the nanosheets. After co-refining the powder diffraction data of the CuFeSe_2_ collected at several energies across the Cu and Fe K-edges and a resonant diffraction experiment, we concluded that the synthesized CuFeSe_2_ crystal is consistent with a tetragonal structure with a slight structural disorder and an Fe-rich composition, as shown in Figure 1b and Table S1. Furthermore, the dominant peak (192.6 cm^−1^) in the Raman spectrum (Figure 1c) is consistent with the tetragonal crystal structure of eskebornite CuFeSe_2_. The STEM-EDS measurement, as shown in Figure 1e–h, shows the uniform distribution of Cu, Fe, and Se elements within the CuFeSe_2_ nanosheets. The TGA plot in Figure S3 shows that the synthesized CuFeSe_2_ nanosheets start to decompose at 524 °C. The weight loss in the range of 100–524 °C is around 3%, corresponding to the evaporation of the residual moisture and the organic ligand originating from the solution-phase synthesis.

**Figure 2:**
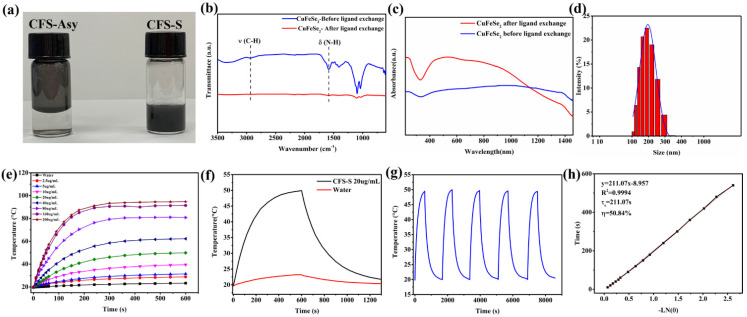
(**a**) Synthesized CuFeSe_2_ nanosheets (1 mg/mL) and ligand-exchanged CuFeSe_2_ (1 mg/mL). (**b**) FTIR spectra. (**c**) UV–Vis–NIR spectra of the synthesized CuFeSe_2_ nanosheets and ligand-exchanged CuFeSe_2_. (**d**) Hydrodynamic diameters of CFS-S. (**e**) Photothermal heating curves of CFS-S aqueous solutions in different concentrations (i.e., 0, 5, 10, 20, 40, 80, and 160 μg/mL) irradiated by an 808 nm laser (1 W/cm^2^, 10 min). (**f**) Heating and cooling curves of CFS-S solution (20 μg/mL) and pure water irradiated with an 808 nm NIR laser for 10 min and then naturally cooled to room temperature. (**g**) Temperature plot of 20 µg/mL CFS-S aqueous solution irradiated by an 808 nm laser (1.0 W/cm^2^) for five on–off cycles. (**h**) Linear time data versus ln (θ) obtained from the cooling period with laser off.

In this work, the oleylamine orientates to the surface of the CuFeSe_2_ crystal through the polar headgroup NH_2_, and the non-polar alkene chain of oleyamine impedes the dispersion of CuFeSe_2_ nanosheets in water [21,22]. As such, an inorganic ion S^2−^ was employed to replace the oleylamine on the surface of the CuFeSe_2_ nanosheets via a ligand exchange process in formamide, resulting in S^2-^-terminated CuFeSe_2_ nanosheets (CFS-S). Typically, nanomaterials prepared through colloidal synthesis usually exhibit metal cation enrichment at their surface, and thus, anionic X-type ligands (-O_2_CR, -Cl, -SR, etc.) are generally used to maintain charge neutrality [23,24,25]. In this work, through colloidal synthesis, the surfaces of the prepared CuFeSe_2_ nanosheets are rich with undercoordinated metal cations, providing numerous electrophilic sites, in turn helping the negatively charged S^2−^ to bind to the surfaces via an ion pair [25]. The negative charging of the CuFeSe_2_ nanosheets after ligand exchange was confirmed with zeta potential. The CFS-S could be dispersed in polar solvents such as water, since it is water-dispersible, as shown in Figure 2a. Compared to the FTIR spectrum (Figure 2b) of the synthesized CuFeSe_2_ nanosheets (blue line), the spectrum of the CFS-S nanosheets (red line) does not show characteristic bands at around 2925 cm^−1^ and 1540–1710 cm^−1^, which are ascribed to the C–H stretching vibration and N–H bending band of oleylamine, respectively, indicating the organic ligand was completely removed. The SEM-EDS analysis of the synthesized CuFeSe_2_ in Figure S4a demonstrates the uniform elemental distribution of Cu, Fe, and Se throughout the nanosheets, and the SEM-EDS map of CFS-S in Figure S4b further proves the presence of the sulfur element in the CFS-S nanosheets. The elemental composition of the synthesized CuFeSe_2_ nanosheets and the S^2−^ terminated CuFeSe_2_ nanosheets (CFS-S) was further examined using XPS. As shown in Figure S5, the XPS spectra of both as-synthesized CuFeSe_2_ nanosheets and the CFS-S nanosheets exhibit Cu 2p with two peaks at 932.4 eV and 952.3 eV, and Fe 2p with two peaks at 711.1 eV and 724.8 eV, suggesting the redox states of Cu and Fe elements to be +1 and +3, respectively [26,27]. Structures related to Se 3d and Se 3p can be observed at around 54 eV, 160.2 eV and 165.8 eV, which is in good agreement with the peaks of Se^2−^ [26,28]. The S 2p with a peak at 162.7 eV presented in the XPS spectrum of CFS-S corresponds to S 2p_1/2_, which is consistent with a valence of −2, proving the presence of S^2−^ in the CFS-S nanosheets [12], where the content of S^2−^ in CFS-S was evaluated to be around 14.6% in atomic percentage.

Figure 2c presents the UV–Vis–NIR absorption spectra of the synthesized CuFeSe_2_ nanosheets and the ligand-exchanged CFS-S. A large absorbance band in the range of 400–1400 nm is observed, which could be attributed to the electronic transitions from the valence band (VB) to the empty intermediate band (IB), where the IB band is mainly composed of Fe 3d orbitals [1,29,30,31]. Furthermore, the extinction coefficient was evaluated by measuring the absorbance of CFS-S with different concentrations at 808 nm (Figure S6), where the absorbance linearly increased with concentration. The calculated extinction coefficient α is 18.38 L g^−1^ cm^−1^.

The size of prepared CFS-S was quantified by DLS, and as shown in Figure 2d, the average hydrodynamic diameter of CFS-S is around 200 nm. The CFS-S aqueous solutions with different concentrations were irradiated continuously with an 808 NIR laser (1 W/cm^2^) for 10 min (Figure 2e). The temperature increments (ΔT) increased with the increase in the CFS-S concentration. For instance, the temperature of the 20 µg/mL CFS-S aqueous solution could rapidly increase from 20 °C to 50 °C within 10 min (Figure 2f), a much faster rate than pure water. Additionally, there is no noticeable attenuation observed (Figure 2g), indicating the excellent photostability of CFS-S. Based on previous reports [18,19] and the in-house-determined temperature–time curve for the 20 µg /mL CFS-S aqueous solution, the photothermal conversion efficiency (η) of CFS-S was calculated to be 50.84%, as shown in Figure 2h.

**Figure 3:**
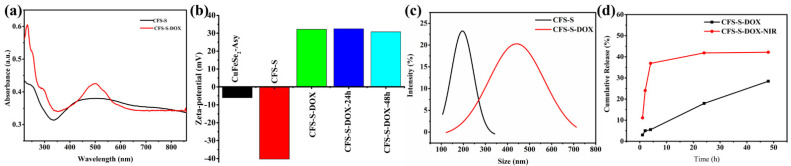
(**a**) UV–Vis spectra before and after CFS-S loading DOX. (**b**) Zeta potential of CuFeSe_2_ NS as-synthesized, CFS-S, CFS-S-DOX, and CFS-S-DOX after laser irradiation and then being held at room temperature for 24 h and 48 h. (**c**) Hydrodynamic diameters of CFS-S (black) and CFS-S-DOX (red). (**d**) Dox release profile of CFS-S-DOX with or without 1.0 W cm^−2^ 808 nm laser (times: 1 h, 2 h, 4 h, 24 h, 48 h).

A commercial chemotherapeutic drug, doxorubicin, was loaded on the surfaces of CFS-S nanosheets to obtain CFS-S-DOX. DOX was noncovalently loaded onto CFS-S by simply stirring for 24 h. Several mixed solutions of CFS-S/DOX were prepared toward maximum loading efficiency, wherein the mass ratios of DOX to CFS-S were 1:1, 1:2, 1:4, 1:8 and 1:16. The amount of DOX grafted on CFS-S nanosheets was estimated by subtracting the amount of unloaded DOX from the initial mass of DOX; the unloaded amount was determined from the absorbance of unloaded DOX in the supernatant, using UV–Vis–NIR spectroscopy. This calculated loading efficiency of DOX is shown in Supplementary Table S2. The maximum loading efficiency was 48.6% when mixing DOX/CFS-S at a mass ratio of 1:2. Further loading validation was achieved by UV–Vis spectroscopy and zeta potential (Figure 3), using the DOX characteristic adsorption band around 500 nm. The zeta potential dramatically changed from −40.5 mV to 32.2 mV due to DOX loading in the CFS-S nanosheets. After grafting DOX onto CFS-S nanosheets, the average hydrodynamic diameter of the resulting CFS-S-DOX increased to around 450 nm, as shown in Figure 3c, which could be attributed to the aggregation of CFS-S-DOX nanostructures.

The irradiation of CFS-S-DOX_(aq)_ at 808 nm (NIR laser, 10 min) did not cause a change in the zeta potential value (~32.5 mV for 24–48 h). The DOX release profile (Figure 3d) shows a change from ~3% with no irradiation to 11% with laser irradiation, within 1 h, and from 28% to 42% after 24 h, showing the photothermal amplification of DOX release.

**Figure 4:**
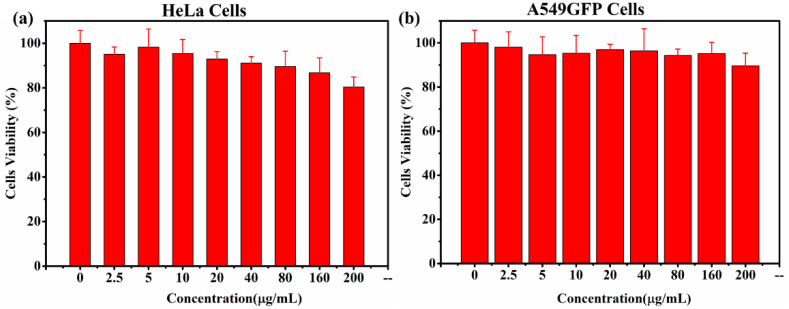
(**a**) Viabilities of HeLa cells and (**b**) A549GFP cells incubated with CFS-S at different concentrations for 24 h.

The biocompatibility of the photothermal agent is essential to ensuring minimal intrinsic toxicity in living cells. A cell viability test (CCK-8 assay) for CFS-S was conducted on HeLa and A549GFP cells, as shown in Figure 4; more than 80% viability was observed at concentrations up to 160 μg/mL.

Optimization experiments showed that increases in laser power density and irradiation time cause cell viability reductions in CFS-S-treated samples, while irradiation alone maintains 98–100% viability, as shown in Figure S7.

**Figure 5:**
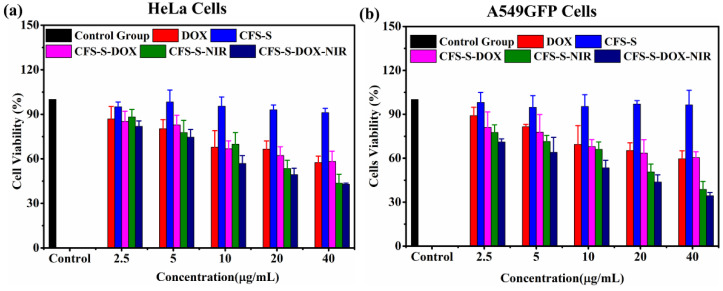
Cytotoxicity assays of (**a**) HeLa cells and (**b**) A549GFP cells with various treatments: CFS-S, DOX, CFS-S upon irradiation, and CFS-S-DOX with or without irradiation.

Cell viability assays were conducted for both HeLa and A549GFP cells with various treatments: CFS-S, DOX, CFS-S with laser irradiation, and CFS-S-DOX with or without laser irradiation (Figure 5a,b). Irradiation with the NIR 808 nm laser led to a remarkable decrease in the cell survival rate compared to CFS-S and CFS-S-DOX without irradiation. Based on the literature, nanoparticles generally enter the cell via the endocytic pathway, which could be divided into two primary categories, including phagocytosis and pinocytosis. Phagocytosis encompasses the uptake of large particles with sizes lager than 250 nm, while pinocytosis is the preferred uptake of small particles with sizes up to 200 nm [16,32,33,34]. In this study, the prepared CFS-S with an average size of ~200 nm could enter the cell via pinocytosis; however, the nanoparticles generally must interact with microenvironment around the cells to form clusters on the surface that lead to an increase of the overall size. Thus, both the CFS-S and CFS-S-DOX in this work were assumed to enter cells through phagocytosis. The cell engulfs the CFS-S or CFS-S-DOX nanosheets in the extracellular matrix via membrane invagination, and then buds off inside the cell to form endocytic vesicles containing CFS-S or CFS-S-DOX, followed by transporting the vesicles to specific subcellular compartments [16,32,33,34]. Nevertheless, it is well known that the physicochemical properties of nanoparticles, such as size, shape, surface charge, surface hydrophobicity/hydrophilicity, and surface functionality, as well as biological factors (such as the macromolecules in the surroundings) and the cell type, all affect the endocytosis pathway and intracellular fate [32,34,35]. Therefore, a thorough analysis of the endocytosis pathway and intracellular fate of CFS-S and CFS-S-DOX needs to be conducted in the future. Herein, confocal microscopy imaging of A549GFP cells treated with CFS-S-DOX with laser irradiation was performed to prove the cell uptake of CFS-S-DOX (Figure S8). The red fluorescence signature of DOX was identified in the cell’s nucleus, indicating the successful DOX release and efficient uptake of CFS-S-DOX by cells.

Generally, under NIR laser irradiation, the photothermal agent, CFS-S, generated localized heat, raising the temperature of its surroundings, which in turn resulted in cell death due to cell necrosis, apoptosis, and necroptosis at high temperatures (>42 °C). According to published reports, the cell necrosis pathway dominates cell death when cells experience temperatures above 49 °C, while necroptosis and apoptosis are induced at around 46 °C [36,37]. The lower cell survival rate of the CFS-S-DOX-NIR group compared to CFS-S-NIR could be ascribed to the enhanced cellular uptake, since the strong thermal effect (>50 °C) induced complete cell necrosis, and a mild photothermal effect (~43 °C) increases the fluidity of the cells’ membrane [15]. Finally, the cell viability of HeLa and A549GFP cells treated with 40 µg/mL of CFS-S-DOX and an 808 nm laser (1 W/cm^2^) approached around 46% and 35%, respectively, which is remarkably lower than the survival rate in the presence of pure DOX, confirming the superior therapeutic efficacy. The ultimate goal of photothermal therapy (PTT) is using an external IR laser to irradiate specific tumor areas that are pretreated with nanosheets, in the hope that the CFS-S-DOX nanosheets loaded to the target tumor sites will provide contribute to the tumor elimination goal.

## 4. Conclusions

We have developed a facile, scalable method to prepare stand-alone 2D CuFeSe_2_ nanosheets via the incorporation of Cu^2+^ cations into FeSe_2_ template nanosheets. The purity of eskebornite CuFeSe_2_ was validated by Raman and XRD, and TEM confirmed the large lateral dimension of the nanosheets, while TGA and UV–Vis–NIR revealed that the synthesized CuFeSe_2_ nanosheets possess relatively high thermal stability and intense absorption in the range of 400–1400 nm, respectively, rendering them suitable for photothermal cancer therapy. The ligand exchange led to CuFeSe_2_ nanosheets capped with S^2−^ (CFS-S), which possess excellent biocompatibility, further confirming their potential for biomedical applications. The photothermal therapy with an 808 nm laser using CFS-S-DOX and CFS-S manifested increases in cancer cell death, compared to laser exposure alone, showing a synergistic therapeutic effect. In conclusion, CuFeSe_2_ nanosheets can be employed as promising photothermal agents.

## Data Availability

The data presented in this study are available on request from the authors.

## Supplementary Materials

The following are available online at https://www.mdpi.com/article/10.3390/nano11082008/s1, Figure S1: XRD pattern (a, left side) and crystal structure (a, right side), Raman spectrum (b), and TEM image (c) of the synthesized FeSe_2_. Figure S2: Synchrotron data for as-synthesized CuFeSe_2_ nanosheets. Figure S3: TGA plot of synthesized CuFeSe_2_ nanosheets. Figure S4: SEM-EDS map of synthesized CuFeSe_2_ nanosheets (a) and CFS-S (b). Figure S5: XPS spectra of (a) as-synthesized CuFeSe_2_ nanosheets and (b) CFS-S nanosheets. Figure S6: UV-Vis-NIR absorption spectra of CuFeSe_2_ solutions in different concentrations (a) and linear fitting curves of the absorbance at 808 nm (b). Figure S7: (a) Cell survival rate of Hela cells cultured with various concentrations of CFS-S nanosheets (0, 10, 20 µg/mL) and then exposed to 808 nm laser with different power density (250 mW/cm^2^ and 1 W/cm^2^); (b) Cell survival rate of Hela cells cultured with various concentrations of CFS-S nanosheets (0 and 10 µg/mL) and then exposed to 808 nm laser (1 W/cm^2^) for different time (0, 1, 3, 5, 10 min). Figure S8: Confocal microscope images of A549GFP cells treated with CFS-S-DOX-NIR (10 µg/mL). Table S1: Crystal structural parameters of as-synthesized CuFeSe_2_ nanosheets. Table S2: Drug Loading Efficiency of DOX to CFS-S.
