# Supplementary material for: Stand-Alone CuFeSe2 (Eskebornite) Nanosheets for Photothermal Cancer Therapy
Source: Nanomaterials (Basel). 2021 Aug 5;11(8):2008. doi: 10.3390/nano11082008 (PMC8398665; doi:10.3390/nano11082008)
Supplement: Supplementary file 1 [file nanomaterials-11-02008-s001.zip › nanomaterials-1328138-supplementary.pdf]

Supplementary Materials

Stand-Alone $CuFeSe_2$ (Eskebornite) Nanosheets for Photothermal Cancer Therapy

Mimi Liu [1], Daniela R. Radu [1], Gurpreet Singh Selopal [2], Saiphaneendra Bachu [3] and Cheng-Yu Lai [1,*]

[1] Department of Mechanical and Materials Engineering, Florida International University, Miami, FL 33199, USA; mliu@fiu.edu (M.L.); dradu@fiu.edu (D.R.R.)
[2] Centre Énergie Materiaux Télecommunications (INRS-EMT), Institut National de la Recherche Scientifique, Varennes, QC J3X 1S2, Canada; gurpreet.selopal@inrs.ca
[3] Department of Materials Science and Engineering, The Pennsylvania State University, University Park, PA 16802, USA; sbachu@psu.edu (S.B.)
* Correspondence: clai@fiu.edu

1. Photothermal conversion efficiency. The photothermal conversion efficiency of CFS-S was evaluated using the following equation:[1,2]

$$\eta = \frac{hS(T_{max} - T_{Surr}) - Q_D}{I(1 - 10^{-A})}$$

where h is the heat transfer coefficient, S the surface area of the container; T_{max} is the equilibrium maximum temperature, T_{Surr} is the surrounding ambient temperature, and Q_D expresses heat dissipated from light absorbed by the solvent and the container. I is the laser power (1 W/cm^2); and A is the absorbance of CFS-S at 808 nm (0.33). Q_D was measured independently to be 28.2 mW using pure water without CFS-S. Herein, hS is derived according to the following equation:

$$\tau_s = \frac{m_D C_D}{hS}$$

where m_D and C_D are the mass (0.3 g) and heat capacity (4.2 J/g) of deionized water, respectively. τ_s is the sample system time constant which can be calculated based on the following equation:

$$t = -\tau_s In(\theta)$$

$$\theta = \frac{T - T_{Surr}}{T_{max} - T_{Surr}}$$

2. The drug (doxorubicin-DOX) loading efficiency. The drug loading efficiency was calculated according to the following equation[3]:

$$Loading\ Efficiency(\%) = \frac{1000MV(C_0 - C_e)}{m} \times 100\%$$

where M is the molar mass of DOX; V is the volume of supernatant; C_0 and C_e are the initial and equilibrium concentrations of DOX; m is the mass of CFS-S.

3. Materials Characterization

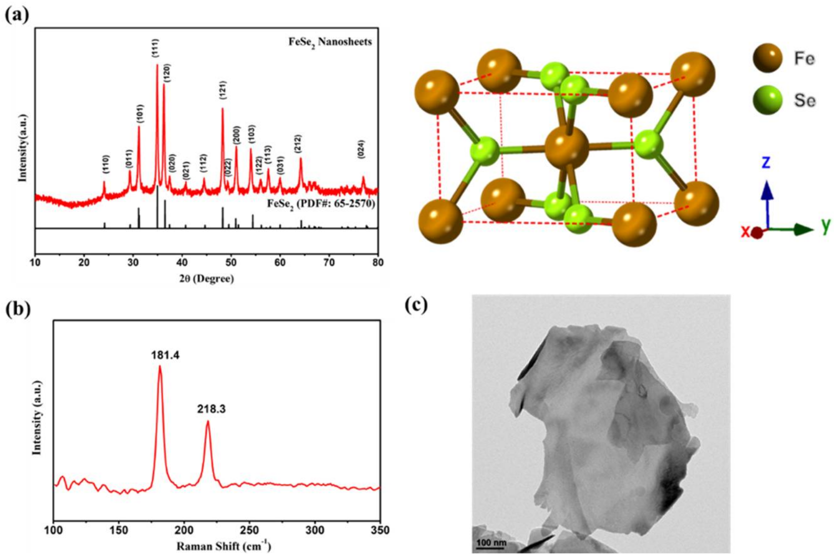

Figure S1. (a) XRD pattern (left) and crystal structure (right) of the synthesized $FeSe_2$ nanosheets. (b) Raman spectrum of the synthesized $FeSe_2$ nanosheets. (c) TEM images of the synthesized $FeSe_2$.

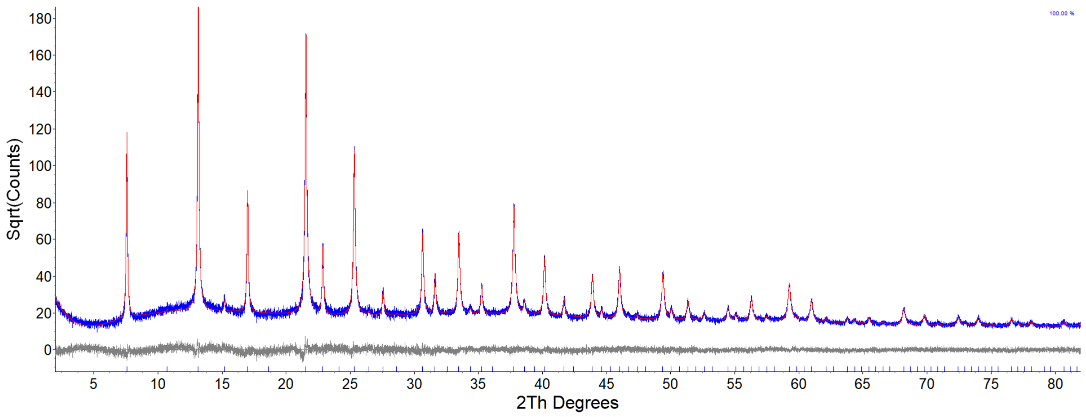

Figure S2. Synchrotron data for as-synthesized $CuFeSe_2$ nanosheets.

Table S1 Crystal structural parameters of as-synthesized $CuFeSe_2$ nanosheets.

Atom Type	X	Y	Z	Occupancy	B (thermal parameter)
Fe	0	0	0	0.904(5)	0.991(1)
Cu	0	0	0	0.096(5)	0.991(1)
Cu	0	0	0.25	0.796(5)	0.991(1)
Fe	0	0	0.25	0.204(5)	0.991(1)
Fe	0	0.5	−0.00224(6)	0.64(1)	0.991(1)
Cu	0	0.5	−0.00224(6)	0.36(1)	0.991(1)
Se	0.2592(1)	0.2461(1)	0.1276(1)	1	0.991(1)

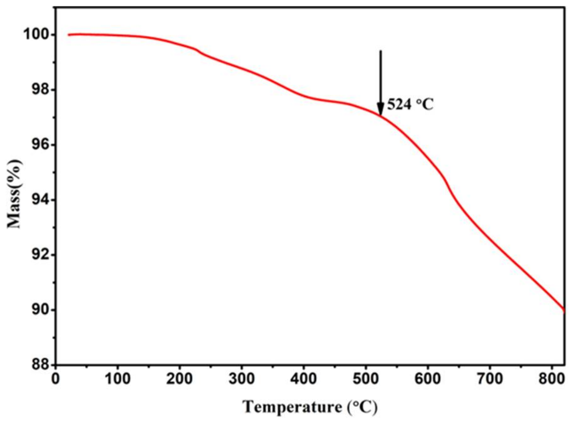

Figure S3 TGA plot of synthesized $CuFeSe_2$ nanosheets.

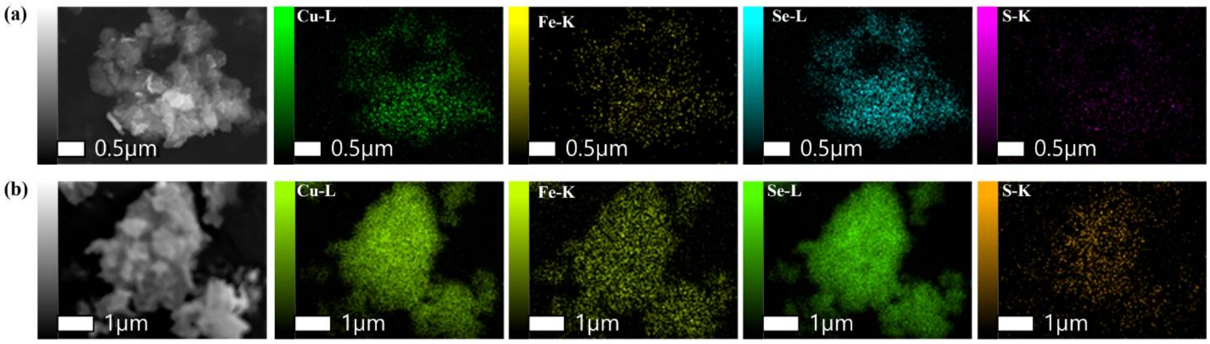

Figure S4. (a) SEM-EDS map of synthesized $CuFeSe_2$ nanosheets. (b) SEM-EDS map of CFS-S.

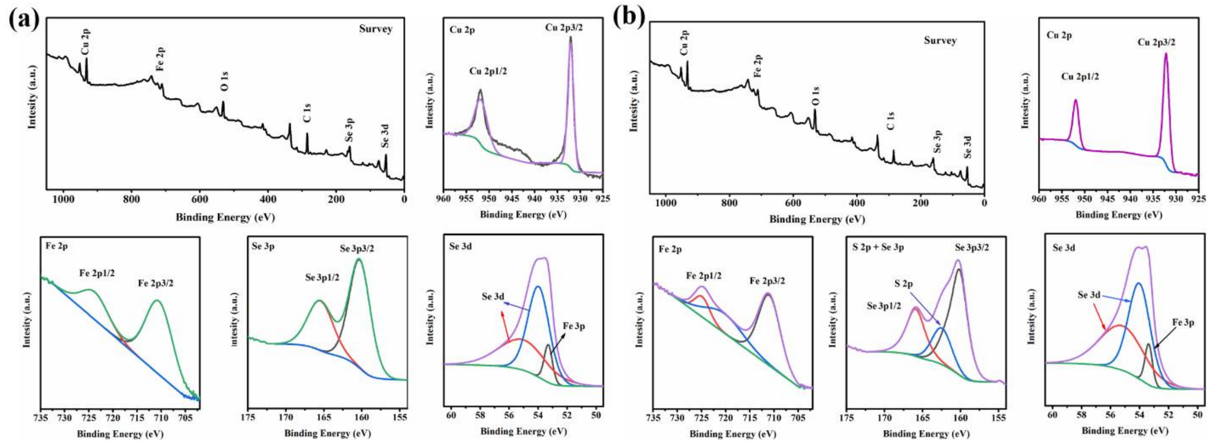

Figure S5 XPS spectra of (a) as-synthesized $CuFeSe_2$ nanosheets, (b) CFS-S nanosheets.

4. Photothermal testing

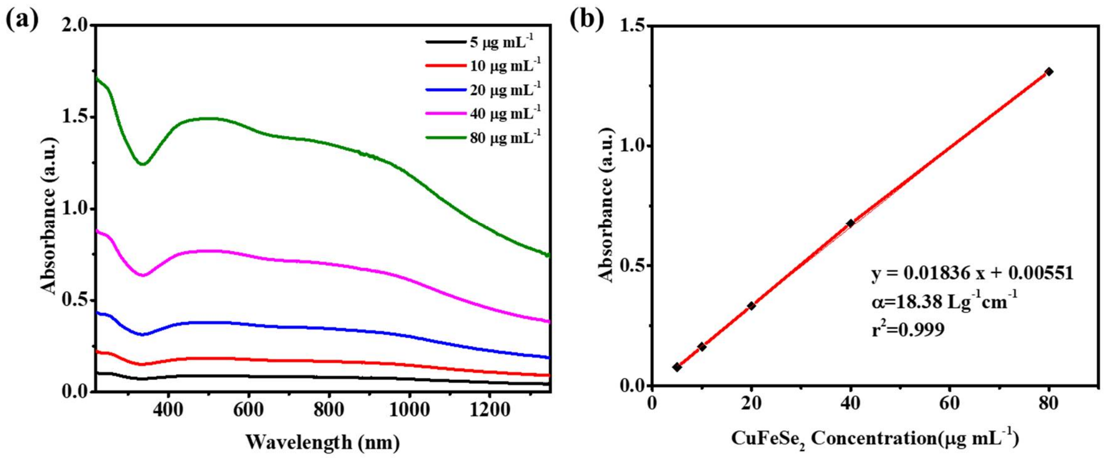

Figure S6. (a) UV-Vis-NIR absorption spectra of $CuFeSe_2$ solutions in different concentrations (i.e., 0, 5, 10 20, 40, and 80 μg mL^{-1}). (b) linear fitting curves of the absorbance at 808 nm.

Table S 2 Drug Loading Efficiency of DOX to CFS-S.

Ratio of DOX: CFS-S	Original DOX (mg)	Original CFS-S (mg)	DOX in supernatant (mg)	Loading Efficiency
1:1	4	4	3.032	24.2%
1:2	2	4	0.056	48.6%
1:4	1	4	0.263	18.5%
1:8	0.5	4	0.142	9.0%
1:16	0.25	4	0.055	4.89%

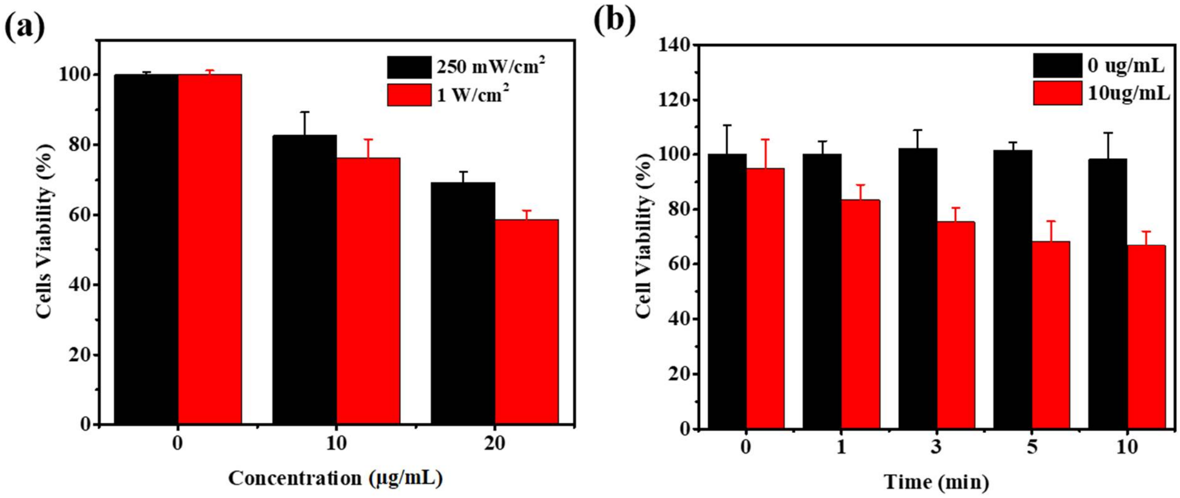

Figure S7. (a) Cell survival rate of Hela cells cultured with various concentrations of CFS-S nanosheets (0, 10, 20 μg/mL) and then exposed to 808 nm laser with different power density(250 mW/cm^2 and 1 W /cm^2) for 10 minutes. (b) Cell survival rate of Hela cells cultured with various concentrations of CFS-S

nanosheets (0 and 10 μg/mL) and then exposed to 808 nm laser (1 W /cm^2) for different time (0, 1, 3, 5, 10 min).

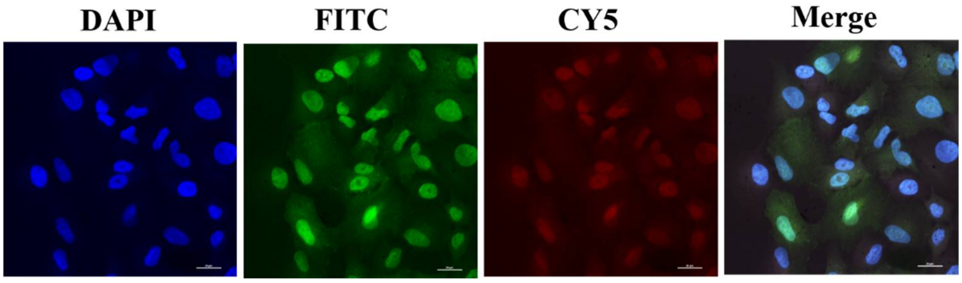

Figure S8. Confocal microscope images of A549GFP cells treated with CFS-S-DOX-NIR (10 μg/mL).

References

(1) Liu, X.; Li, B.; Fu, F.; Xu, K.; Zou, R.; Wang, Q.; Zhang, B.; Chen, Z.; Hu, J. Facile synthesis of biocompatible cysteine-coated CuS nanoparticles with high photothermal conversion efficiency for cancer therapy. *Dalton Transactions* **2014**, *43*, 11709-11715.

(2) Tian, Q.; Hu, J.; Zhu, Y.; Zou, R.; Chen, Z.; Yang, S.; Li, R.; Su, Q.; Han, Y.; Liu, X. Sub-10 nm Fe_3O_4@$Cu_{2-x}S$ Core–Shell Nanoparticles for Dual-Modal Imaging and Photothermal Therapy. *Journal of the American Chemical Society* **2013**, *135*, 8571-8577.

(3) Yin, W.; Yan, L.; Yu, J.; Tian, G.; Zhou, L.; Zheng, X.; Zhang, X.; Yong, Y.; Li, J.; Gu, Z.; Zhao, Y. High-Throughput Synthesis of Single-Layer MoS2 Nanosheets as a Near-Infrared Photothermal-Triggered Drug Delivery for Effective Cancer Therapy. *ACS Nano* **2014**, *8*, 6922-6933.
